# Perceived Role Responsibility, Self‐Efficacy, and Education Needs of School Nurses Providing Mental Health Services: A Nationwide Survey

**DOI:** 10.1111/josh.70210

**Published:** 2026-08-02

**Authors:** Hsing‐Yi Yu, Chun‐Hsia Huang, Yung‐Chao Shen, Hui‐Ling Lin, Li‐Chun Chang

**Affiliations:** ^1^ School of Nursing, College of Medicine, Chang Gung University Taoyuan Taiwan; ^2^ Department of Nursing New Taipei Municipal TuCheng Hospital New Taipei Taiwan; ^3^ Department of Nursing Linkou Branch, Chang Gung Memorial Hospital Taoyuan Taiwan; ^4^ School of Nursing, Chang Gung University of Science and Technology Taoyuan Taiwan; ^5^ Taipei Medical University Taipei Taiwan

**Keywords:** continuing education, mental health, perceived role responsibility, school nurses, self‐efficacy, Taiwan

## Abstract

**Background:**

School nurses increasingly support student mental health, yet how perceived role responsibility and self‐efficacy shape continuing education needs remains unclear.

**Methods:**

A nationwide cross‐sectional online survey was conducted in Taiwan using probability proportional to size sampling. Among 658 selected school nurses, 305 responded (46.4%). A 33‐item questionnaire assessed perceived role responsibility, self‐efficacy, and continuing education needs across six competency domains. Multivariable linear regression identified predictors.

**Results:**

Most nurses lacked prior psychiatric/psychology work experience (74.4%). Perceived role responsibility and self‐efficacy were highest for referral/resource mapping and crisis response/safety assessment, and lowest for psychotropic medication management/monitoring. Continuing education needs were high, particularly for crisis response/safety assessment and identification/assessment. Perceived role responsibility was the only significant predictor of continuing education needs, explaining 29.4% of the variance.

**Implications for School Health Policy, Practice, and Equity:**

Training should prioritize role‐focused interventions and standardized protocols for crisis response/safety assessment and identification/assessment, with clear medication‐related coordination guidance to reduce role ambiguity.

**Conclusions:**

School nurses' perceived role responsibility drives continuing education needs; training should strengthen role clarity alongside skills.

## 1 Introduction

According to the World Health Organization, adolescent mental health issues significantly impact global health, with one in seven individuals aged 10–19 affected by mental health conditions. Depression, anxiety, and behavioral disorders are among the leading causes of illness and disability in this age group. Additionally, suicide is the third leading cause of death among youths aged 15–29 years [1]. Since the onset of the COVID‐19 pandemic, mental health concerns among students have steadily increased, driven in part by academic pressure and social media use [2]. Early identification and nonpharmacological interventions can help prevent the progression of mental illness while minimizing the risks of overtreatment and institutionalization [3].

School nurses play a crucial role in the early identification of mental health issues in children and adolescents [4] and in providing school‐based mental health interventions [5, 6]. However, the delivery of such support is inherently shaped by the staffing and regulatory context in which nurses operate. In Taiwan, while the “one‐school–one‐nurse” policy under the School Health Act mandates at least one nurse per campus to ensure essential health services [7, 8], the actual nurse‐to‐student ratio varies markedly because staffing is determined by class numbers rather than the number of students. A recent national survey revealed that each Taiwanese school nurse cared for an average of 812.86 students (SD = 777.48) [9], and approximately 40%–50% of schools had a nurse‐to‐student ratio greater than 1:750 [10]. Although Taiwanese school nurses provide services and functions broadly comparable to those described in other countries, these roles should be understood within Taiwan's distinct education system, policy environment, and staffing model.

To contextualize these mental health service expectations, school nurses have been described in the international literature as providing a wide range of mental health services, including direct care, case management, interprofessional collaboration, referrals, mental health screenings, and outreach efforts such as mental health education [11]. However, the implementation of these services tends to vary both between and within districts, particularly in areas such as suicide prevention [12]. Given that school nurses fulfill multiple roles in school mental health services, they need additional resources and knowledge to meet service demands [2, 13]. School nurses' perceived role responsibility for specific mental health service components, such as coordinating psychotropic medication monitoring and participating in suicide risk assessment and counseling, may be particularly important for interdisciplinary collaboration [14]. Previous studies indicate that school nurses need training on supporting students with mental health issues and collaborating with teachers, administrators, and external agencies (e.g., community mental health centers and psychiatric outpatient services); in addition, the need for training in anxiety, attention deficit hyperactivity disorder, substance use disorders, behavioral challenges, and therapeutic communication has been highlighted [15, 16]. Crucially, insufficient training has been shown to limit the scope and effectiveness of the care they can provide [11]. Although these findings primarily reflect Western practices, such training gaps and resource demands are highly relevant to Taiwanese school nurses, who operate under a comparable scope of practice but face distinct localized constraints.

Despite growing attention to school nurses' mental health responsibilities and training gaps, the determinants of their continuing education needs in mental health service provision remain underexplored. Prior studies suggest that lower perceived capability to deliver mental health care and lower professional self‐efficacy are associated with gaps in mental health preparedness and training [17, 18]. These gaps may in turn undermine the effectiveness and coping capacity of school nurses in practice [11, 19]. In addition, uncertainty about the inclusion of mental health services within the scope of their responsibilities indicates that perceived role responsibility for mental health service provision also warrants attention [20]. Perceived role responsibility refers to the extent to which school nurses agree that providing specific components of school‐based mental health services should be part of their professional role, including screening, counseling, referral, collaboration, and psychiatric medication‐related care. Self‐efficacy refers to perceived confidence in performing mental health–related tasks, such as assessment, referral, crisis response, school‐based interventions, and psychotropic medication management.

Therefore, this study aims to examine school nurses' perceived role responsibility, self‐efficacy, and continuing education needs in mental health service provision and to identify factors associated with these needs. This study addresses the identified research gaps by employing a quantitative survey approach. The findings can help inform the development of evidence‐based educational programs and policy strategies to enhance the capacity and quality of mental health services provided by school nurses.

## 2 Methods

### 2.1 Design

This cross‐sectional study used data from a nationwide online survey in Taiwan and is part of a broader research series examining mental health services provided by school nurses. Previous publications have presented findings related to existing service provision [21], and this study expands on those findings by exploring additional dimensions, specifically focusing on school nurses' perceived role responsibility, self‐efficacy, and continuing education needs related to mental health services. This report was prepared in accordance with the Strengthening the Reporting of Observational Studies in Epidemiology (STROBE) guidelines for cross‐sectional studies [22].

### 2.2 Participants

According to statistical data from Taiwan's Ministry of Education, the country had 4304 schools as of August 2024 [23]. Sample size estimation for multivariable analysis was conducted using G*Power 3.1.9.7 software. Based on a linear regression model with 18 predictors, an effect size of 0.1, power of 0.95, and significance level (alpha) of 0.01, the minimum required sample size was 392 participants [24]. Considering the school nurse response rate of 59.5% reported in a previous study [25], at least 658 questionnaires would be needed to achieve the desired sample size.

To enhance the representativeness of the sample, probability proportional to size sampling was applied using the 2024 school directory from the Ministry of Education. The sampling frame included full‐time nurses employed in elementary, junior high, senior high/vocational schools, and universities/colleges. Six hundred and fifty‐eight potential participants were identified based on the relative proportion of school nurses in each county and school level, and participants were randomly selected within each stratum using the random function in Excel.

The inclusion criteria were full‐time, licensed school nurses currently employed at K–12 schools or higher education institutions, thereby excluding those working in kindergartens or special education institutions, or employed in part‐time or temporary roles.

### 2.3 Instrumentation

A structured questionnaire was used to assess the participants' demographic characteristics, perceived role responsibility, self‐efficacy, and continuing education needs related to providing mental health services.

#### 2.3.1 Demographic Characteristics

The collected data included age, marital status, education level, years of experience as a school nurse, school type, geographic region, student‐to‐nurse ratio, employment type (permanent or substitute nurse), and prior clinical experience in mental health settings (e.g., psychiatry or mental health nursing).

#### 2.3.2 Perceived Role Responsibility for Providing Mental Health Services

Perceived role responsibility for providing mental health services was measured using a 12‐item scale adapted from items reported in the NASN Weekly Electronic Digest on school nurses' current mental health screening practices, perceived barriers, and related practices [26]. In this study, these items were revised to assess school nurses' perceived role responsibility for providing specific components of school‐based mental health services. The scale included items such as providing mental health screenings, psychological counseling, cognitive behavioral interventions, substance/alcohol abuse interventions, referrals to school and community mental health professionals, discussions with parents/guardians, organizing mental health promotional activities, participating in crisis teams, providing consultations for school staff, and managing or monitoring psychiatric medications. For example, one item asked whether “providing mental health screenings” should be part of the school nurse's role in delivering school‐based mental health services. Items were rated on a 5‐point Likert scale (1 = strongly disagree to 5 = strongly agree), with higher scores indicating stronger perceived role responsibility for providing specific components of school‐based mental health services. Content validity was confirmed, with all items showing an item‐level content validity index > 0.75 and a scale‐level content validity index/average of 0.93. Cronbach's alpha for this scale was 0.93 in this study.

#### 2.3.3 Self‐Efficacy in Delivering Mental Health Services

Self‐efficacy was assessed using a self‐developed 6‐item scale informed by the Mental Health Training Intervention for Health Providers in Schools framework developed by the National Center for School Mental Health and the National Association of School Nurses [27]. The items evaluated confidence in (1) strategies for promoting positive interactions related to mental health, (2) identification and assessment, (3) referrals and resource mapping, (4) crisis response and safety assessment, (5) best practices for school‐based interventions, and (6) psychotropic medication management. Items were rated on a 5‐point Likert scale (1 = not confident at all to 5 = very confident), with higher scores indicating greater self‐efficacy. Content validity was supported, with all items achieving an item‐level content validity index > 0.75 and a scale‐level content validity index/average of 0.92. Cronbach's alpha was 0.94 in this study.

#### 2.3.4 Continuing Education Needs for Mental Health Services

This self‐developed 6‐item scale, informed by the Mental Health Training Intervention for Health Providers in Schools framework [27], measured the degree of need for continuing education across the same six domains as the self‐efficacy scale. Responses were rated on a 5‐point Likert scale (1 = not needed at all to 5 = very much needed). Higher scores indicate a greater perceived need for continuing education. The scale demonstrated strong content validity (content validity index = 0.92) and high internal consistency (Cronbach's alpha = 0.96).

### 2.4 Procedure

Data were collected using an online questionnaire distributed via Google Forms. Contact information for school nurses was obtained from the School Directory of the Ministry of Education and official institutional websites, and invitation emails containing the survey link were sent to school nursing units. All participants received an information sheet detailing the study's purpose, procedures, and their rights, including voluntary participation, anonymity, and the right to withdraw at any time without penalty. Participation was voluntary, and consent was obtained at the start of the survey; only those who agreed could proceed with the questionnaire. Because the study collected anonymous self‐reported data on school nurses' perceptions, additional institutional consent was not required. Respondents received a small gift voucher (NT$50) at the time of invitation, regardless of survey completion. To protect data security, survey responses were stored in a password‐protected cloud‐based account accessible only to the first author, minimizing unauthorized access. No personally identifiable information (e.g., names or email addresses) was collected, and all responses were anonymous. The first author was solely responsible for data management and analysis. Upon completion of data collection, the online responses were exported into an Excel file and subsequently converted into an SPSS dataset for statistical analysis.

This study was approved by the Institutional Review Board of the first author's affiliated institution (Approval Date: June 24, 2024; IRB No. 202400915B0). All data were used solely for research purposes and reported in aggregate form.

### 2.5 Data Analysis

Data were analyzed using SPSS Statistics version 28 (IBM Corp., Armonk, NY, USA). Descriptive statistics were used to summarize the participants' demographics, perceived role responsibility, self‐efficacy, and continuing education needs related to the provision of mental health services. In the analyses, perceived role responsibility, self‐efficacy, and continuing education needs were represented by the mean scores of their respective scales. To identify the factors associated with continuing education needs, bivariate analyses were initially conducted to screen for significant variables. These significant variables were subsequently entered into a stepwise multiple regression model to identify the predictors.

Two sensitivity analyses were conducted. First, to address the probability proportional to size sampling design, an additional analysis was conducted using the Complex Samples procedures in SPSS. Sampling strata were defined by region and school level, and sampling weights were calculated based on the ratio of the population size to the valid sample size within each stratum. Complex Samples general linear modeling was used to examine whether the main regression findings were robust after accounting for stratification and sampling weights. Second, to assess the robustness of the findings within the K–12 context, a sensitivity analysis was conducted after excluding the subsample of school nurses from higher education institutions (*n* = 39). A *p*‐value < 0.05 was considered statistically significant.

## 3 Results

### 3.1 Participant Characteristics

The online survey was administered to 658 potential participants, yielding 320 completed questionnaires, of which 305 were valid (response rate of 46.4%) (Table 1). The average age of participants was 47.57 (SD = 7.18) years, with the majority under the age of 50 and married. Over half held a bachelor's degree, and two‐thirds had more than 10 years of professional experience as a school nurse. Most participants worked in elementary schools, with more than one‐third of the school nurses serving in Northern Taiwan. More than half of the participants worked in schools with a nurse‐to‐student ratio exceeding 1:750. In addition, 227 participants (74.4%) reported no prior clinical experience in psychiatric nursing.

**TABLE 1:** Demographic characteristics of nurses in schools and higher education institutions (*N* = 305).

Variable	*n*	%
Age (mean = 47.57, SD = 7.18)		
< 50	178	58.4
> 50	127	41.6
Marital status		
Unmarried	32	10.5
Married	247	81.0
Divorced	20	6.5
Widowed	6	2.0
Educational level		
Junior college	50	16.4
Undergraduate	169	55.4
Graduate school	86	28.2
School experience, years (mean = 12.99, SD = 7.73)		
< 10	103	33.8
> 10	202	66.2
School setting		
Elementary school	167	54.8
Junior high school	57	18.6
Senior high school	42	13.8
University	39	12.8
Service region		
Northern Taiwan	108	35.4
Central Taiwan	92	30.1
Southern Taiwan	81	26.6
Eastern Taiwan	24	7.9
Nurse‐to‐student ratio		
> 1:750	166	54.4
< 1:750	139	45.6
Employment type		
Substitute	7	2.3
Full time	298	97.7
Clinical experience in psychology		
Yes	78	25.6
No	227	74.4

Abbreviation: SD, standard deviation.

### 3.2 Perceived Role Responsibility and Self‐Efficacy in Providing Mental Health Services

Participants reported relatively stronger perceived role responsibility for facilitating referrals to school and community mental health professionals and participating in crisis teams. Conversely, they reported relatively lower perceived role responsibility for managing and monitoring psychiatric medications within mental health service provision (Table 2).

**TABLE 2:** Perceived role responsibility of school nurses in providing mental health services (*N* = 305).

	Item	Mean^a^	SD
	Total	3.48	0.83
1	Providing mental health screenings	3.41	1.04
2	Psychological counseling	3.18	1.13
3	Cognitive behavioral interventions	3.21	1.10
4	Substance/alcohol abuse interventions	3.25	1.12
5	Referrals to school mental health professionals	4.27	0.88
6	Referrals to community mental health professionals	4.06	0.96
7	Discussions with parents/guardians about students' mental health issues	3.58	1.10
8	Organize mental health promotion activities	3.49	1.12
9	Participating in school mental health crisis teams	3.81	0.89
10	Providing mental health consultations for school staff	3.54	1.07
11	Managing psychiatric medications	2.82	1.30
12	Monitoring psychiatric medication use	3.07	1.25

Abbreviation: SD = standard deviation.

^a^ Mean scores ranged from 1 to 5, with higher scores indicating stronger perceived role responsibility for providing mental health services.

Regarding self‐efficacy, the participants reported the highest confidence in “Mental health referrals and resource mapping” and “Mental health crisis response and safety assessment.” “Psychotropic medication management” displayed the lowest self‐efficacy (Table 3). Continuing education needs were reported across all areas, with the greatest demand for training in “Mental health crisis response and safety assessment” and “Identification and assessment of mental health issues.” “Psychotropic medication management” received lower continuing education need ratings than other areas (Table 3).

**TABLE 3:** Self‐efficacy and continuing education needs in delivering mental health services of school nurses (*N* = 305).

	Item	Self‐efficacy		Continuing education needs	
		Mean^a^	SD	Mean^b^	SD
	Total	2.80	0.88	4.02	0.84
1	Strategies and skills for promoting positive and supportive interactions regarding students' mental health issues	2.73	0.99	4.06	0.91
2	Identification and assessment of mental health issues	2.87	0.97	4.08	0.92
3	Mental health referrals and resource mapping	2.95	1.03	4.06	0.87
4	Mental health crisis response and safety assessment	2.89	1.00	4.13	0.89
5	Practices for mental health interventions	2.75	0.96	4.01	0.94
6	Psychotropic medication management	2.68	1.10	3.82	1.07

Abbreviation: SD, standard deviation.

^a^ Mean scores range from 1 to 5, with higher scores indicating greater self‐efficacy.

^b^ Mean scores range from 1 to 5, with higher scores indicating greater perceived need for continuing education.

### 3.3 Associations Between Participant Characteristics, Perceived Role Responsibility, and Self‐Efficacy and Continuing Education Needs

Bivariate and multiple linear regression analyses were conducted to identify factors associated with continuing education needs (Table 4). In the bivariate analysis, the significant predictors included working in Eastern Taiwan (*B* = 0.426, *t* = 2.394, *p* < 0.05), self‐efficacy in delivering mental health services (*B* = 0.221, *t* = 4.124, *p* < 0.001), and perceived role responsibility for mental health service provision (*B* = 0.558, *t* = 11.346, *p* < 0.001).

**TABLE 4:** Bivariate and multiple linear regression analyses of continuing education needs related to mental health services among school nurses (*N* = 305).

Variables	*β*	Bivariate			Multivariate			Δ*R* ^2^
	95% CI	*t*	*R* ^2^	*β*	95% CI	*t*
Age (reference: < 50)	−0.077	−0.270–0.116	−0.787	0.002				
Marital status (reference: Married)								
Unmarried	0.136	−0.174–0.446	0.863	0.009				
Divorced	0.197	−0.187–0.581	1.011	0.003				
Widowed	0.372	−0.312–1.057	1.070	0.004				
Educational level (reference: Undergraduate)								
Junior college	−0.116	−0.373–0.140	−0.892	0.003				
Graduate school	0.166	−0.045–0.377	1.552	0.008				
School experience of year (reference: > 10 years)	−0.191	−0.391–0.009	−1.877	0.011				
School setting (reference: Elementary school)								
Junior high school	−0.098	−0.342–0.146	−0.789	0.002				
Senior high school	0.244	−0.031–0.519	1.744	0.010				
University	0.012	−0.273–0.297	0.080	0.000				
Service region (reference: Northern Taiwan)								
Central Taiwan	0.103	−0.104–0.310	0.979	0.003				
Southern Taiwan	−0.075	−0.290–0.141	−0.683	0.002				
Eastern Taiwan	0.426	0.076–0.777	2.394*	0.019	0.131	−0.172‐0.434	0.849	
Nurse‐to‐student ratio (reference:> 1:750)	−0.084	−0.275–0.107	−0.868	0.002				
Employment type (reference: Full time)	0.268	−0.367–0.903	0.831	0.002				
Clinical experience in psychology (reference: No)	0.018	−0.236–0.200	0.162	0.000				
Self‐efficacy in delivering mental health services	0.221	0.116–0.327	4.124***	0.050	0.030	−0.068‐0.129	0.607	
Perceived role responsibility for providing mental health services	0.558	0.461–0.655	11.346***	0.303	0.539	0.435‐0.644	10.136***	0.294

Abbreviations: B, unstandardized regression coefficient; CI, confidence interval; Δ*R* ^2^, change coefficient of determination.

* *p* < 0.05.

*** *p* < 0.001.

In the multiple linear regression analysis, perceived role responsibility for mental health service provision remained a significant positive predictor of continuing education needs (*B* = 0.539, *t* = 10.136, *p* < 0.001), accounting for 29.4% of the variance (Δ*R* ^2^ = 0.294) while age, marital status, educational level, school experience, school setting, substitute nurse status, and clinical experience in psychology were not significant predictors. Self‐efficacy, which was significant in the bivariate analysis, was not significant in the multivariate model. Similarly, the significance of the Eastern Taiwan service region in the bivariate analysis diminished in the multivariate model.

### 3.4 Sensitivity Analysis

The complex samples sensitivity analysis yielded results consistent with the primary analysis. After accounting for sampling strata and weights, perceived role responsibility for mental health service provision remained a significant predictor of continuing education needs (*B* = 0.604, SE = 0.068, 95% CI [0.471, 0.737], *p* < 0.001), whereas self‐efficacy and Eastern Taiwan service region were not significant predictors (Table 5).

**TABLE 5:** Complex samples sensitivity analysis predicting continuing education needs (*N* = 305).

Variables	*B*	SE	95% CI	Wald *F*	*p*
Intercept	1.964	0.322	[1.331, 2.598]		< 0.001
Service region: Eastern Taiwan	−0.087	0.108	[−0.301, 0.126]	0.650	0.421
Perceived role responsibility for providing mental health services	0.604	0.068	[0.471, 0.737]	79.782	< 0.001
Self‐efficacy in delivering mental health services	0.017	0.062	[−0.105, 0.139]	0.075	0.785

*Note:* Complex Samples General Linear Model was conducted using sampling strata defined by region and school level and sampling weights based on the population‐to‐sample ratio within each stratum. The dependent variable was continuing education needs. Wald *F* is not reported for the intercept.

Abbreviations: *B*, unstandardized regression coefficient; SE, standard error; CI, confidence interval.

After excluding school nurses from higher education institutions, the sensitivity analysis yielded results consistent with the primary findings. Both the bivariate and multivariable analyses showed a similar pattern to that observed in the full sample. Specifically, perceived role responsibility for providing mental health services remained the only significant predictor of continuing education needs (*B* = 0.573, *t* = 9.957, *p* < 0.001), accounting for 32.3% of the variance. Detailed results are provided in Table S1.

## 4 Discussion

Continuing education may serve as an important means for school nurses to adapt to the significant shifts in mental health service provision in the post‐pandemic era. It may also help sustain confidence [28] and enhance the competence of school nurses in addressing the evolving mental health needs of students [29].

Notably, 74.4% of the school nurses in this study reported having no prior work experience in psychiatry or psychology. School nurses often transition from hospital‐based nursing roles, in which their experience typically focuses on physical care in areas such as internal medicine, surgery, or critical care. Consequently, they often have limited backgrounds in psychiatric or psychological care, highlighting the need for specialized mental health training [30].

School nurses frequently identify referring students to mental health professionals and community resources as an important aspect of their role. This aligns with extensive research highlighting that as frontline healthcare providers, their role within interprofessional mental health teams, particularly in facilitating referrals to resources, is a critical component of mental health service delivery [26, 30]. Studies indicate that such referrals represent a direct mental health service provided by school nurses, whether for general mental health screening or for students identified as being at high risk. Previous studies suggest that such referrals may contribute to improvements in students' mental well‐being [31].

School nurses generally perceived participation in school mental health crisis teams as a critical component of their professional responsibilities. However, in practice, they continue to face numerous challenges in interprofessional collaboration. For example, within school systems, nurses are not consistently regarded as natural partners or resource providers for addressing students' mental health concerns [30]. Additionally, variations in school and district policies and resource allocation have led to unequal opportunities for school nurses to participate in interdisciplinary collaboration [12]. Factors contributing to these disparities include the exclusivity of interprofessional collaboration [32], a limited nursing workforce [12], a lack of clear and structured guidelines for interventions [33], geographic and resource limitations [29], and inadequate education and professional training [34]. Therefore, although school nurses highly value and express a strong willingness to engage in interdisciplinary collaboration, they may not always have the requisite conditions or opportunities to meaningfully participate in such practices [30]. Furthermore, in many countries, crisis management policies, such as those for student suicide prevention, feature clearly defined roles and procedural guidelines (e.g., school safety mechanisms and reporting systems) [31]. These structured frameworks may contribute to stronger perceived role responsibility for these tasks among school nurses.

Prior research has consistently shown that school nurses often report low self‐efficacy in delivering mental health services [12, 18, 28, 33], and our findings align with this pattern. In this study, psychotropic medication management received the lowest self‐efficacy scores and was also rated lower than other domains in perceived role responsibility and continuing education needs. Because analyses did not test for statistical domain‐specific associations, these findings are interpreted as descriptive observations rather than inferred correlations. Nevertheless, psychotropic medication management received relatively lower scores across these measures, indicating that it may be a less emphasized area in the current school nursing context in Taiwan.

These findings contrast with those of Bohnenkamp et al. [26], who reported that in the United States, administering and monitoring psychotropic medications are commonly recognized components of school nurses' mental health practices. In Taiwan, however, psychotropic medication management appears more constrained by structural and regulatory conditions, including limited policy support and unclear guidance, the absence of on‐site school physicians, and underdeveloped referral and feedback linkages with specialist mental health resources [9]. In addition, ambiguity regarding roles and responsibilities for medication‐related tasks may heighten concerns about legal accountability and clinical risk, further limiting the feasibility of such practices in schools [35]. Consequently, even when medication‐related needs arise in practice, psychotropic medication management may be less likely to be viewed as a core component of the school nurse role. This may help explain why this domain received lower ratings for school nurses' perceived role responsibility, self‐efficacy, and continuing education needs. Taken together, these findings suggest that school nurses' engagement in mental health service provision may vary across practice domains, with stronger preparedness in referral‐ and crisis‐related areas than in psychotropic medication management.

More broadly, these results indicate that school nurses reported substantial continuing education needs, suggesting an unmet demand for training in student mental health care [18, 30]. Similar gaps in role clarity and capacity have been reported among school nurses in the United States, such as in Washington, DC, where school nurses described limited capacity to support behavioral health programs [36]. Although a recent scoping review indicates that pre‐licensure nursing curricula in many countries, including Taiwan, typically incorporate foundational mental health content [37], evidence from a US‐based school nursing intervention suggests that sustained, evidence‐based professional development opportunities may further enhance mental health competencies [28].

Currently, continuing education for school nurses in mental health care lacks consistency and continuity [19], and opportunities for targeted mental health training remain limited [27]. In this study, school nurses working in Eastern Taiwan reported significantly higher continuing education needs in the bivariate analysis, which may reflect regional differences in access to educational resources. These challenges may be particularly relevant in geographically remote areas, where access to continuing education resources and professional development opportunities may be more limited. According to Skundberg‐Kletthagen and Moen [30], a variety of training formats can offer valuable learning experiences for school nurses, considering the unique demands of their roles. Even short‐term mental health courses have been reported to help school nurses address adolescent mental health challenges effectively. In addition, online curriculum focusing on mental health topics [38] and simulated scenarios [28] have demonstrated the potential to support school nurses' mental health practices. However, the literature on structured mental health training programs specifically designed for school nurses remains limited. Future research should prioritize the development and evaluation of evidence‐based, contextually relevant mental health training interventions tailored to the school nursing workforce [19], particularly for nurses working in underserved areas.

Among all areas of continuing education needs, mental health crisis response, safety assessment, and the identification and assessment of mental health issues received the highest ratings. This pattern may be related to the central role of assessment and screening in school nursing practice [26]. Conducting mental health–related assessments and screenings remains a natural extension of school nurses' clinical roles, as these practices enable the early identification of high‐risk students and facilitate timely referrals to appropriate mental health services [39]. However, this role orientation may also be associated with how school nurses are positioned within the school system, which may limit their recognition as integral mental health partners and the perceived value of their broader contributions in this domain [30].

Although self‐efficacy in providing mental health services is described as an important factor in continuing education [29], the regression analysis revealed that upon including school nurses' perceived role responsibility for mental health services in the model, perceived role responsibility emerged as the only significant predictor. Considering the broad scope of school nurses' responsibilities, their service priorities may also be influenced by national‐, local‐, and school‐level policies, particularly in the context of multilayered changes to school health services caused by the COVID‐19 pandemic [40]. Therefore, understanding school nurses' perceived role responsibility for mental health service provision may be important for designing targeted training programs that align with real‐world service demands. These findings suggest that training programs should not only build skills but also strengthen school nurses' perceived role responsibility and clarity in mental health service provision.

### 4.1 Implications for School Health Policy, Practice, and Equity

School nurses are well positioned to support school‐based mental health through their clinical training and daily presence in schools. In this study, perceived role responsibility for mental health service provision emerged as a factor associated with continuing education needs, and psychotropic medication–related coordination and monitoring received relatively low self‐efficacy scores. These findings suggest that professional development may benefit from the inclusion of role‐focused components and clearer scope‐of‐practice guidance. Training may also need to address areas in which school nurses reported lower self‐efficacy. In addition, schools may benefit from strengthening triage, referral, and follow‐up pathways to support consistent mental health services across campuses with unequal access to mental health professionals.

### 4.2 Limitations

This study had several limitations. First, it employed a cross‐sectional design to examine school nurses' perceived role responsibility, self‐efficacy, and continuing education needs related to mental health services. Therefore, caution should be exercised when interpreting causal relationships. Second, all participants in this study were female, and the data were collected exclusively in Taiwan. Given differences in mental health policies and nursing practices across countries and regions, the generalizability of the findings to other contexts may be limited. Third, although data collection was scheduled to avoid peak academic periods, the response rate was 46.4%. Given that response rates in school nurse surveys over the past decade have often been modest, this level of participation was relatively strong and fell within the higher range reported in recent studies [41, 42, 43, 44]. Nevertheless, nonparticipation may still have introduced response bias; future research should examine reasons for nonresponse and implement strategies to enhance participation and representativeness. Fourth, although the final sample size did not meet the minimum target estimated in the a priori power analysis, the study still offers useful nationwide data. However, the findings should be interpreted with caution because the study may have had limited power to detect smaller effects. Fifth, using a mean total score for continuing education needs allowed us to examine overall perceived educational needs, but it may have reduced sensitivity to differences across specific content areas. Future research may benefit from analyzing continuing education needs by individual content domain. Finally, this study did not collect data on prior mental health–related training experiences, which limited our ability to directly assess the gap between perceived educational needs and received training. Future studies should include various types of mental health training and evaluate nurses' perceptions of the effectiveness of these programs in enhancing their competencies.

## 5 Conclusion

This study examined school nurses' perceived role responsibility, self‐efficacy, and continuing education needs related to mental health services in Taiwan. Referral to mental health professionals and community resources received relatively higher ratings, whereas psychiatric medication management and monitoring received lower ratings across perceived role responsibility and self‐efficacy, as well as the lowest rating for continuing education needs. Perceived role responsibility for mental health service provision was the only significant predictor of continuing education needs. These findings suggest that school nurses' perceived role responsibility is an important consideration in planning continuing education and support for mental health services.

## Ethics Statement

Ethical approval was granted by the Chang Gung Medical Foundation Institutional Review Board (approval number: 202400915B0, approval time: June 24, 2024).

## Conflicts of Interest

The authors declare no conflicts of interest.

## Supporting information


**Table S1:** Demographic characteristics of nurses in schools and higher education institutions (*N* = 266).
**Table S2:** The perceived role responsibility of school nurses in providing mental health services (*N* = 266).
**Table S3:** Self‐efficacy and continuing education needs in delivering mental health services of school nurse (*N* = 266).
**Table S4:** Bivariate and multiple linear regression analyses of continuing education needs related to mental health services among school nurses (*N* = 266).

## Data Availability

The data that support the findings of this study are available on request from the corresponding author. The data are not publicly available due to privacy or ethical restrictions.
