# Supplementary material for: Perceived Role Responsibility, Self‐Efficacy, and Education Needs of School Nurses Providing Mental Health Services: A Nationwide Survey
Source: J Sch Health. 2026 Aug 2;96(8):e70210. doi: 10.1111/josh.70210 (PMC13429283; doi:10.1111/josh.70210)
Supplement: Supplementary file 1 — Table S1: Demographic characteristics of nurses in schools and higher education institutions (N = 266). Table S2: The perceived role responsibility of school nurses in providing mental health services (N = 266). Table S3: Self‐efficacy and continuing education needs in delivering mental health services of school nurse (N = 266). Table S4: Bivariate and multiple linear regression analyses of continuing education needs related to mental health services among school nurses (N = 266). [file JOSH-96-0-s001.docx]

Table S1 Demographic characteristics of nurses in schools and higher education institutions (N = 266)

| Variable | n | % |
| --- | --- | --- |
| Age (mean=47.57, SD=7.18) |  |  |
| <50 | 145 | 54.5 |
| >50 | 121 | 45.5 |
| Marital status |  |  |
| Unmarried | 28 | 10.5 |
| Married | 212 | 79.7 |
| Divorced | 20 | 7.5 |
| Widowed | 6 | 2.3 |
| Educational level |  |  |
| Junior college | 46 | 17.3 |
| Undergraduate | 151 | 56.8 |
| Graduate school | 69 | 25.9 |
| School experience, years (mean=12.99, SD=7.73) |  |  |
| <10 | 79 | 29.7 |
| >10 | 187 | 70.3 |
| School setting |  |  |
| Elementary school | 167 | 62.8 |
| Junior high school | 57 | 21.4 |
| Senior high school | 42 | 15.8 |
| Service Region |  |  |
| Northern Taiwan | 95 | 35.7 |
| Central Taiwan | 77 | 29.0 |
| Southern Taiwan | 74 | 27.8 |
| Eastern Taiwan | 20 | 7.5 |
| Nurse-to-student ratio |  |  |
| >1:750 | 164 | 61.7 |
| <1:750 | 102 | 38.3 |
| Employment type |  |  |
| Substitute | 7 | 2.6 |
| Full time | 259 | 97.4 |
| Clinical experience in psychology |  |  |
| Yes | 67 | 25.2 |
| No | 199 | 74.8 |

Note. SD, standard deviation

Table S2 The perceived role responsibility of school nurses in providing mental health services (N =266)

|  | Item | Mean | SD† |
| --- | --- | --- | --- |
|  | Total | 3.50 | 0.81 |
| 1 | Providing mental health screenings | 3.44 | 1.04 |
| 2 | Psychological counselling | 3.23 | 1.11 |
| 3 | Cognitive behavioural interventions | 3.25 | 1.08 |
| 4 | Substance/alcohol abuse interventions | 3.26 | 1.12 |
| 5 | Referrals to school mental health professionals | 4.22 | 0.90 |
| 6 | Referrals to community mental health professionals | 4.02 | 0.95 |
| 7 | Discussions with parents/guardians about students’ mental health issues | 3.63 | 1.09 |
| 8 | Organize mental health promotion activities | 3.52 | 1.12 |
| 9 | Participating in school mental health crisis teams | 3.82 | 0.88 |
| 10 | Providing mental health consultations for school staff | 3.65 | 0.97 |
| 11 | Managing psychiatric medications | 2.85 | 1.26 |
| 12 | Monitoring psychiatric medication use | 3.11 | 1.22 |

Note. †SD, standard deviation

Table S3. Self-efficacy and continuing education needs in delivering mental health services of school nurse (N =266)

|  |  | Self-efficacy | | Continuing education needs | |
| --- | --- | --- | --- | --- | --- |
|  | Item | Mean^a^ | SD† | Mean^b^ | SD† |
|  | Total | 2.78 | 0.87 | 4.02 | 0.85 |
| 1 | Strategies and skills for promoting positive and supportive interactions regarding students’ mental health issues | 2.73 | 0.98 | 4.08 | 0.90 |
| 2 | Identification and assessment of mental health issues | 2.86 | 0.96 | 4.10 | 0.91 |
| 3 | Mental health referrals and resource mapping | 2.88 | 1.02 | 4.04 | 0.88 |
| 4 | Mental health crisis response and safety assessment | 2.87 | 0.98 | 4.12 | 0.90 |
| 5 | Practices for mental health interventions | 2.73 | 0.96 | 4.02 | 0.93 |
| 6 | Psychotropic medication management | 2.63 | 1.09 | 3.78 | 1.09 |

Note. ᵃMean scores range from 1 to 5, with higher scores indicating greater self-efficacy; ᵇMean scores range from 1 to 5, with higher scores indicating greater perceived need for continuing education; †SD, standard deviation.

Table S4. Bivariate and multiple linear regression analyses of continuing education needs related to mental health services among school nurses (N = 266).

|  |  | **Bivariate** |  |  | **Multivariate** | | |  |
| --- | --- | --- | --- | --- | --- | --- | --- | --- |
| **Variables** | Β | 95 % C.I. ^‡^ | t† | R^2^ | Β | 95 % C.I. ^‡^ | t† | △R^2^ |
| Age (reference: < 50) | -.084 | -.290-.121 | -0.808 | .002 |  |  |  |  |
| Marital status (reference: Married) |  |  |  |  |  |  |  |  |
| Unmarried | .154 | -.179-0.488 | .912 | .003 |  |  |  |  |
| Divorced | .201 | -.187-.589 | 1.020 | .004 |  |  |  |  |
| Widowed | .375 | -.314-1.064 | 1.071 | .004 |  |  |  |  |
| Educational level (reference: Undergraduate) |  |  |  |  |  |  |  |  |
| Junior college | -.111 | -.381-.160 | -.804 | .002 |  |  |  |  |
| Graduate school | .230 | -.002-463 | 1.954 | .014 |  |  |  |  |
| School experience of year (reference: >10 years) | -.003 | -.016-.011 | -0.364 | .001 |  |  |  |  |
| School setting (reference: Elementary school) |  |  |  |  |  |  |  |  |
| Junior high school | -.099 | -.349-.150 | -.785 | .002 |  |  |  |  |
| Senior high school | .251 | -.028-531 | 1.770 | .012 |  |  |  |  |
| Service region (reference: Northern Taiwan) |  |  |  |  |  |  |  |  |
| Central Taiwan | .133 | -.093-.358 | 1.159 | .005 |  |  |  |  |
| Southern Taiwan | -.097 | -.325-.132 | -.834 | .003 |  |  |  |  |
| Eastern Taiwan | .489 | .105-.873 | 2.507* | .023 | .119 | -.210-.448 | .711 |  |
| Nurse-to-student ratio (reference:> 1: 750) | -.111 | -.321-.100 | -1.037 | .004 |  |  |  |  |
| Employment type (reference: Full time) | .268 | -.372-.907 | .824 | .003 |  |  |  |  |
| Clinical experience in psychology (reference: No) | .016 | -.220-.253 | .137 | .000 |  |  |  |  |
| Self-efficacy in delivering mental health services | .224 | .109-.388 | 3.852*** | .053 | .024 | -0.081-.128 | .447 |  |
| Attitude towards providing mental health services | .590 | .486-.694 | 11.184*** | .321 | .573 | .459-.686 | 9.957*** | .323 |

Note.†: **p* < .05, ***p* < .01, *** *p* < .001; ‡: C.I = confidence interval; ΔR² = change coefficient of determination ; B = unstandardized regression coefficient
